# Identification and Functional Analysis of EPOR^+^ Tumor-Associated Macrophages in Human Osteosarcoma Lung Metastasis

**DOI:** 10.1155/2020/9374240

**Published:** 2020-08-18

**Authors:** Yanxing Li, Ming Li, Rong Wei, Junlong Wu

**Affiliations:** ^1^Department of Orthopedic Surgery, Luoyang Central Hospital Affiliated to Zhengzhou University, Luoyang 471009, China; ^2^Department of Orthopedic and Surgery, The Affiliated Cancer Hospital of Zhengzhou University, Zhengzhou University, Zhengzhou 450001, China

## Abstract

**Background:**

Tissue-resident macrophages can be educated to tumor-associated macrophages (TAMs) by the tumor microenvironment and many types of macrophages express erythropoietic receptor (EPOR); However, little is known about the expression of EPOR on TAMs and the identity of EPOR^+^ TAMs in osteosarcoma lung metastasis has thus far remained elusive.

**Methods:**

EPOR-eGFPcre mice were used to determine the expression of EPOR on lung tissue-resident macrophages. Flow cytometry, RT-PCR, and Western blot were examined to define the identity of EPOR^+^ TAMs in 106 osteosarcoma lung metastasis specimens. Moreover, the clinicopathologic factors and prognosis of patients with CD163^+^EPOR^+^ macrophages were compared.

**Results:**

We found that a subpopulation of mouse lung tissue-resident macrophages express EPOR and EPO enhances the proliferation of EPOR^+^ macrophages in mouse lung. A subpopulation of CD163^+^ macrophages expresses EPOR in human osteosarcoma lung metastasis specimens. CD163^+^EPOR^+^TAMs increase 2.5 times in human osteosarcoma lung metastasis tissues; CD206, CD163, and PD1, which are known to have a significant role in TAM function had high expression in CD163^+^EPOR^+^ TAMs compared with CD163^+^EPOR^−^ TAMs. Furthermore, CD163^+^EPOR^+^ TAMs had higher M2 marker and cytokine expression in osteosarcoma tissues compared with para-osteosarcoma tissues. EPO enhanced the expression of M2 cytokines in primary CD163^+^EPOR^+^ TAMs. Importantly, the percentage of CD163^+^EPOR^+^ TAMs had a positive linear association with malignant phenotypes as well as poor disease-free survival and overall survival time.

**Conclusions:**

We have characterized TAMs expressing EPOR and CD163^+^EPOR^+^ macrophages as TAMs in osteosarcoma lung metastasis patients, which are highly associated with tumor aggressiveness.

## 1. Introduction

Osteosarcoma is the most common type of bone tumor and mainly occurs in young patients, with a peak incidence of 18 years [1, 2]. Despite the development of multimodality therapies such as surgery, systemic therapy, and immunotherapy, the prognosis for osteosarcoma remains poor [3–6]. The prognosis and patient survival are strongly correlated with the treatment response and metastatic status. The lungs are the most common site of metastasis in patients with osteosarcoma postoperatively [7]. The 5-year survival rate is approximately 70% and 20% in patients with no metastases and lung metastases, respectively [8]. Therefore, an understanding of pathophysiology underlying osteosarcoma lung metastases is a prerequisite for future improvement in therapeutic approaches.

Tumor-associated macrophages (TAMs) have anti-inflammatory characteristics or are selectively activated toward the M2 phenotype, which express CD163 [9], CD206 [10], PD1 [11], CD14 [12], and CD16 [13]. Additionally, TAMs secrete anti-inflammatory cytokines (referred to as M2 cytokines herein) such as interleukin- (IL-) 10, transforming growth factor- (TGF-) *β*1, insulin growth factor- (IGF-) 1, and vascular endothelial growth factor A (VEGFA), which can promote cancer progression by different mechanisms [14–16]. None of the abovementioned molecules alone are sufficiently distinctive to determine real TAMs. Most of osteosarcoma patients have anemia, which can induce high levels of erythropoietin (EPO) [17]. EPO and the EPO receptor (EPOR) are indispensable to erythropoiesis [18, 19]. Additionally, angiogenesis, neuroprotection, heart development, and apoptotic cell clearance have also been reported for the contribution of the EPO-EPOR system. Previous studies have demonstrated that EPOR is expressed in nonerythroid cells, such as the brain, kidneys, heart, and various tumor cell lines [20–24]. Specifically, it has been reported that EPOR can be expressed by macrophages in bone marrow (BM), spleen (SP), fetal liver (FL), liver, and peritoneum, which are responsive to EPO [24–27]. CD163 is the main marker on TAMs and is associated with a poor prognosis in patients with cancer [28, 29]. Little is known, however, about the expression of EPOR on TAMs and the identity of CD163^+^EPOR^+^ TAMs in osteosarcoma lung metastasis has yet to be fully explored.

We hypothesized that TAMs express EPOR, which is a main component of TAMs, and EPO enhances the protumor functions of EPOR^+^ macrophages to promote the progression of osteosarcoma lung metastasis. We tested this hypothesis using EPOR-eGFPcre knock in mice and human osteosarcoma lung metastasis samples in vivo and in vitro. We demonstrated that nearly all the lung tissue-resident macrophages expressed EPOR-eGFP in mouse, which can respond to EPO. We also showed that CD163^+^EPOR^+^ TAMs were increased 2.5-fold in human osteosarcoma lung metastasis samples. CD163^+^EPOR^+^ TAMs expressed higher CD206, CD163, and PD1, which are known to have a significant role in the TAM function. Additionally, the percentage of CD163^+^EPOR^+^ TAMs had a positive linear association with malignant phenotypes including the number of lung metastases, the maximal diameter of lung metastases, and pathologic grade, as well as poor disease-free survival (DFS) and overall survival (OS), especially among patients with anemia. In conclusion, our study suggests that TAMs in osteosarcoma lung metastasis samples express EPOR and CD163^+^EPOR^+^ macrophages are true TAMs that can promote the progression of osteosarcoma lung metastasis under EPO stimulation. Improving anemia and targeting CD163^+^EPOR^+^ TAMs may serve as potential therapeutic interventions in osteosarcoma lung metastasis.

## 2. Materials and Methods

### 2.1. Antibodies

All antibodies used in this study are listed in Supplementary Table 1.

### 2.2. Mice and Associated Experiments

In this study, wild type (WT) mice and EPOR-eGFPcre knock in mice [27, 30] were used and maintained at Zhengzhou University Animal Facility. All animal protocols are approved by the Institutional Animal Care and Use Committee. These two types of mice are C57BL/6 background. Adult mice (8-12 weeks old) were used. Mice received an intraperitoneal injection of bromodeoxyuridine (BrdU; 200 ul (10 mg/ml)), as previously described [31]. Mice were sacrificed 24 h after the BrdU injection with anesthesia. Mice were injected with EPO as previously described [32] and on day 8, mice were sacrificed with anesthesia; Lungs were obtained and handled as previously described [33]. Briefly, lungs were isolated from mice, cut into pieces, and incubated in 10 ml warm digestion buffer ((containing Hanks balanced salt solution, 3.26 mM CaCl_2_, 40 mM HEPES, 2 U/ml DNase I grade II (Roche, Barce, Switzerland), and 0.2% Collagenase type IV (Solarbio, Shanghai, China)) for 30 min. Then, the cells were washed twice and gently pushed through a 70 um cell strainer. After centrifugation at 100 g for 3 min, the supernatant was collected. Then, the supernatant was washed once and a single cell suspension was obtained. The cells were subjected to flow cytometry. For flow cytometry, AF647-F4/80 (2 ul in 1 × 10^6^ cells), APC-Cy7-Gr-1(1 ul in 1 × 10^6^ cells), BV-605-CD45 (0.4 ul in 1 × 10^6^ cells), and PE-Cy7-CD11b (2 ul in 1 × 10^6^ cells) were used. For sorting, AF-647-F4/80 and APC-Cy7-Gr-1 were used. For BrdU detection, lung single cell suspensions were fixed and permeabilized by following BD Fix Buffer I (Cat#: 557870, BD bioscience, USA) and Perm Buffer III (Cat#: 558050, BD bioscience, USA) protocols. Then, the cells were stained with APC-Cy7-Gr-1, AF647-F4/80, anti-GFP antibody (2 ul in 1 × 10^6^ cells), and AF-700-anti-BrdU antibody (2 ul in 1 × 10^6^ cells). During the staining process, purified rat anti-mouse CD16/32 (Cat#: 553142, 1: 100, BD bioscience, USA) was used to block nonspecific binding. Finally, cells were run on a Fortessa (BD bioscience, USA). For the negative control, fluorescence minus one (FMO) was used to gate the positive and negative populations. All the mouse experiments were repeated independently at least six times. Flow Jo software was used to analyze the data.

### 2.3. Patients

Fresh lung metastatic osteosarcoma and para-osteosarcoma tissues (obtained 2-3 cm from osteosarcoma tissues) were obtained intraoperatively from patients with osteosarcoma lung metastases; specimens were only obtained from patients with osteosarcoma lung metastases after the first surgery combined with neoadjuvant or/and adjuvant chemotherapy who were eligible to undergo surgery of lung metastatic sites in the Affiliated Cancer Hospital of Zhengzhou University. We enrolled 106 patients undergoing surgical resection of lung metastases between January 2016 and June 2017. After surgery, all the patients received standard chemotherapy. Clinical and postoperative follow-up data were collected. The DFS was calculated from the date of surgery to the time of recurrence or metastasis. The OS was calculated from the date of surgery to the time of death as previously described [34, 35]. If the patients were still alive at the last contact, OS was defined as the time from surgery to the time of last contact.

### 2.4. Single Cell Preparation for Sorting and Flow Cytometry Analysis

Single cells from lung metastatic osteosarcoma and para-osteosarcoma specimens were prepared as previously described [27, 36]. Briefly, the osteosarcoma and para-osteosarcoma tissues were minced and resuspended the same digestion buffer as mentioned above. The suspension was incubated at 37°C for 30 min. After incubation, the suspension was diluted 1 : 1 with Iscove's Modified Dulbecco's Medium (IMDM, Hyclone, USA) and gently pushed through 70 *μ*m cell strainers. The collection was centrifuged at 100 g for 3 min, and the single-cell supernatant was transferred to a new 50 ml tube. Then, the single-cell suspensions were divided into two fractions.

CD14^+^ cells were isolated from one fraction according to the Human CD14-positive selection kit protocol (Miltenyi Biotec, USA). Fluorescence-activated cell sorting- (FACS-) based purification of EPOR^+^ macrophages in osteosarcoma and para-osteosarcoma tissues was performed by staining with APC-CD163 and PE-EPOR. The stained and sorting methods are the same to recently published [27]. Sorted cells were used to perform RT-PCR and western blot to determine the expression of IL10, TGFB1, VEGFA, and IGF1.

The second fraction was stained with APC-Cy7-CD45 (1 ul in 1 × 10^6^ cells), APC-CD163 (2 ul in 1 × 10^6^ cells), PE-EPOR (4 ul in 1 × 10^6^ cells), BV510-CD206 (1 ul in 1 × 10^6^ cells), Percp-CD14 (1 ul in 1 × 10^6^ cells), PE-Cy7-CD16 (1 ul in 1 × 10^6^ cells), BV786-CD80 (4 ul in 1 × 10^6^ cells), and BV605-PD1 (4 ul in 1 × 10^6^ cells) to determine the identity of CD163^+^EPOR^+^ and CD163^+^EPOR^−^ macrophages. At the same time, the expression of CD206, CD163, PD1, CD14, and CD16 was also determined between osteosarcoma and para-osteosarcoma tissues. During the process of staining, PBS plus 0.4% human AB serum was used to block nonspecific binding as previously described [27, 37–39]. Finally, cells were run on a Fortessa (BD bioscience, USA). For the negative control, FMO was used to gate the positive and negative populations. Flow Jo software was used to analyze the data. To determine the percentage of CD163^+^EPOR^+^ and CD163^+^EPOR^−^ macrophages in CD45^+^ cells, we costained the cells with CD45, CD163, and EPOR and subjected the stained cells to flow cytometry to determine the ratio of CD163^+^EPOR^+^ and CD163^+^EPOR^−^ in CD45^+^ cells in osteosarcoma and para-osteosarcoma tissues.

### 2.5. EPO Induced Phosphorylation of Macrophages and Total Proteins Extraction

Primary macrophages from osteosarcoma patients were cultured for 24 h by using IMDM plus 1%FBS to starve macrophages. Then, added EPO to stimulate macrophages for 10 minutes. After EPO stimulation, harvested the adherent macrophages by scraper as well as the control adherent macrophages. Total proteins were extracted, and protein concentration was measured as previously described [27, 37–40].

### 2.6. EPO Induced mRNA Changes in Macrophages

The adherent macrophages were cultured for 24 h by using IMDM plus 1%FBS to starve macrophages. Then, add EPO to stimulate macrophages for another 24 h. Total RNA was isolated from adherent macrophages, concentration was determined, and reverse transcript to cDNA finally. The detailed methods were the same to our previous papers [27, 37–40].

Quantitative real-time PCR (qRT-PCR) qRT-PCR was examined by using SYBR™ Select Master Mix (Thermo Fisher Scientific, USA) and primer pairs of interest, with human ACTB as the internal control. The primer pairs were designed using Harvard primer bank and synthesized by Eurofins MWG Operon LLC. The primer sequences are listed in Supplementary Table 2. Results are representative of three independent experiments.

### 2.7. Western Blot

By using our prepared proteins, WB did as previously described [27, 37–40]. The following antibodies were used: P-STAT5 (1 : 1000 dilution), STAT5 (1 : 1000 dilution), P-AKT (1 : 1000 dilution), AKT (1 : 1000 dilution), IL10 (1 : 1000 dilution), TGFB1 (1 : 1000 dilution), VEGFA (1 : 1000 dilution), IGF1 (1 : 1000 dilution), and goat anti-rabbit IgG-HRP secondary antibody (1 : 5000 dilution), Blots were developed on GeneSnap enhanced chemiluminescence system (SynGene) by using an ECL Western Blotting Substrate (BeyoECL Plus, Beyotime Institute of Biotechnology). Results are representative of at least three independent experiments.

### 2.8. Statistical Analysis

GraphPad Prism 7.0 software was used for statistical analysis. DFS and OS were calculated by Kaplan-Meier, and the prognostic factors were analyzed by univariate and multivariable analyses. Other data used *t*-test. For all statistical analyses, significance is indicated as at least *P* < 0.05.

## 3. Results

### 3.1. A Subpopulation of Mouse Lung Tissue-Resident Macrophages Express EPOR-eGFP

**Figure 1:**
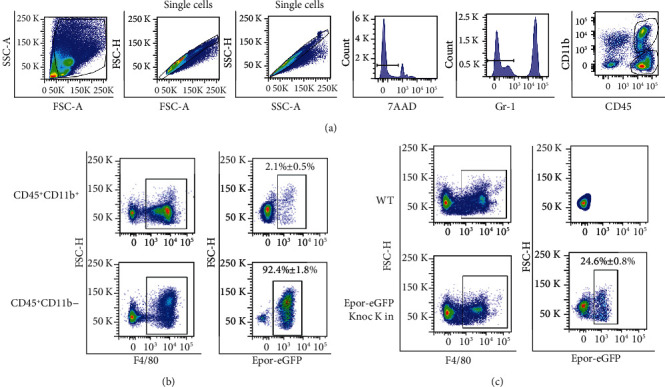
A subpopulation of mouse lung tissue-resident macrophages express EPOR-eGFP. (a) Representative flow cytometry analysis of macrophages in mouse lung, *N* = 6. (b) Representative and quantitative analysis EPOR-eGFP expression in CD45^+^F4/80^+^CD11b^−^ and CD45^+^F4/80^+^CD11b^+^ macrophages, *N* = 6; (c) Representative flow cytometry and quantitative analysis of EPOR-eGFP percentage in lung F4/80^+^ macrophages, WT mice were used as control, *N* = 6.

Previous studies indicated that lung-tissue resident macrophages are CD45^+^F4/80^+^CD11b^low/-^ [41]. We first examined whether tissue-resident macrophages expressed EPOR in mouse lungs. To test this, we used an EPOR-eGFPcre knock in mouse model, which was described in a recent publication [27]. Then, we stained lung mononuclear cells (MNCs) from EPOR-eGFPcre mice using Gr1, F4/80, CD45, and CD11b antibodies. Figures 1(a) and 1(b) show that >90% of CD45^+^F4/80^+^CD11b^−^macrophages (lung-tissue resident macrophages) are EPOR-eGFP^+^, while only 2% of CD45^+^F4/80^+^CD11b^+^ macrophages are EPOR-eGFP^+^. Based on this observation, we developed a further analysis of F4/80^+^EPOR-eGFP^+^ and F4/80^+^EPOR-eGFP^−^ macrophages.  Figure 1(c) indicates that in mouse lungs, approximately 25% of F4/80^+^ macrophages expressed EPOR-eGFP. At the same time, WT mice were used as negative controls, no GFP signals were detected in WT F4/80^+^ macrophages. Our data indicate that a subpopulation of mouse lung tissue-resident macrophages expressed EPOR.

### 3.2. EPO Injection Led to Increased EPOR^+^ Lung Tissue-Resident Macrophage Infiltration by Increasing Proliferation

**Figure 2:**
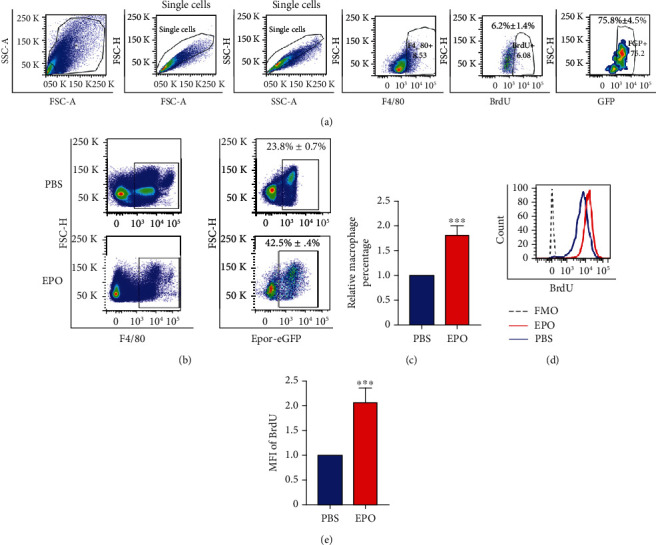
EPO injection led to increased EPOR^+^ lung tissue-resident macrophage infiltration by increasing proliferation. (a) Representative flow cytometry analysis and quantitative analyses GFP (EPOR) expression on F4/80^+^BrdU^+^ macrophages in the lung. *N* = 6. (b) Representative flow cytometry analysis of EPOR-eGFP percentage in lung F4/80^+^ macrophages after EPO injection, PBS injection as control, *N* = 6. (c) Quantitative analyses of F4/80^+^EPOR^+^ macrophages in lung after EPO injection, *N* = 6. (d) Representative flow cytometry analysis of BrdU expression in lung F4/80^+^EPOR-eGFP^+^ macrophages after EPO injection, *N* = 6. Dotted line: FMO (e) quantitative analyses of mean BrdU expression in lung F4/80^+^EPOR-eGFP^+^ macrophages after EPO injection, *N* = 6.

Having shown that a subpopulation of mouse lung tissue-resident macrophages expressed EPOR-eGFP, a hallmark of tissue-resident macrophages is the ability to self-renew. To test this property of tissue-resident macrophages, we injected BrdU into EPOR-eGFPcre mice; lung MNCs were stained with F4/80, anti-BrdU, and anti-GFP antibodies. Flow cytometry (Figure 2(a)) showed that after BrdU injection, only about 6% of F4/80^+^ macrophages expressed BrdU. At the same time, mice without BrdU injection were used as negative controls, FMO data showed that no BrdU was detected in F4/80^+^EPOR-eGFP^+^ macrophage (Supplementary Figure 1A). Interestingly, to further analyze F4/80^+^BrdU^+^ macrophages, we found that >75% of F4/80^+^BrdU^+^ macrophages were EPOR-eGFP^+^. Supplementary Figure 1B shows that EPOR-eGFP FMO had no signals in F4/80^+^BrdU^+^ macrophages. This result suggested that EPO enhanced the proliferation of EPOR-eGFP^+^ macrophages in the lungs. Then, we determined whether EPO increased the percentage of EPOR-eGFP^+^ macrophages in the lungs after EPO treatment. After EPO injection, the percentage of F4/80^+^EPOR-eGFP^+^ macrophages increased >1.5-fold in lungs (Figures 2(b) and 2(c)). To further confirm the proliferation of F4/80^+^EPOR-eGFP^+^ macrophages after EPO injection, BrdU was injected into mice after EPO injection, Figures 2(d) and 2(e) indicate that EPO injection increased the proliferation of EPOR-eGFP^+^ macrophages; however, the proliferation of EPOR-eGFP^−^ macrophages did not change (data not shown). Taken together, EPOR^+^ lung tissue-resident macrophages responded to EPO and the proliferative capacity increased after EPO injection.

### 3.3. The Percentage of CD163^+^EPOR^+^ Lung Macrophages Increased in Osteosarcoma Lung Metastases Specimens

**Figure 3:**
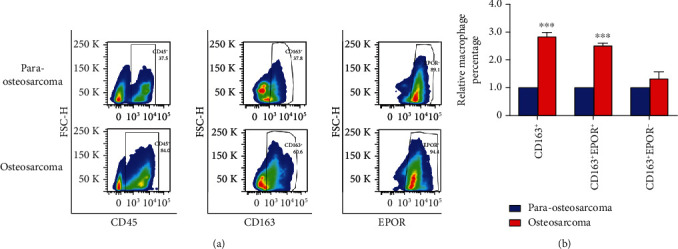
Examine and quantitative CD163^+^EPOR^+^ macrophages and CD163^+^EPOR^−^ macrophages in osteosarcoma lung metastasis specimens. (a) Representative flow cytometry analysis of CD163^+^EPOR^+^macrophages in osteosarcoma tissues and para-osteosarcoma tissues *N* = 106. (b) Quantitative analyses of CD163^+^EPOR^+^macrophages and CD163^+^EPOR^−^ macrophages in osteosarcoma tissues and para-osteosarcoma tissues. *N* = 106.

We showed that a subpopulation of mouse lung tissue-resident macrophages express EPOR-eGFP. To determine whether macrophages express EPOR in human lung metastasis, fresh osteosarcoma and para-osteosarcoma tissue specimens were obtained from 106 curative resection patients with lung metastasis and single cells were prepared for flow cytometry. CD45, CD163, and EPOR were stained.  Figure 3(a) shows the representative flow cytometry images from osteosarcoma and para-osteosarcoma specimens, which indicated that a subpopulation of CD163^+^ macrophages expressed EPOR. Quantitative analysis indicated that the percentage of CD163^+^ macrophages in CD45^+^ cells from osteosarcoma tissues increased 2.8-fold compared with para-osteosarcoma tissues in 106 cases (Figure 3(b)). To further analyze the percentage of macrophages, we showed that the percentage of CD163^+^EPOR^+^ macrophage increased 2.5-fold; however, there were no significant differences in the percentage of CD163^+^EPOR^−^ macrophages between osteosarcoma and para-osteosarcoma tissues. Together, these findings clearly demonstrated that a subpopulation of human osteosarcoma lung metastasis macrophages expressed EPOR and CD163^+^EPOR^+^ macrophages increased in osteosarcoma lung metastases specimens.

### 3.4. M1/M2 Molecules Had Different Expression on CD163^+^EPOR^+^ and CD163^+^EPOR^−^ Macrophages in Osteosarcoma Lung Metastasis Specimens

**Figure 4:**
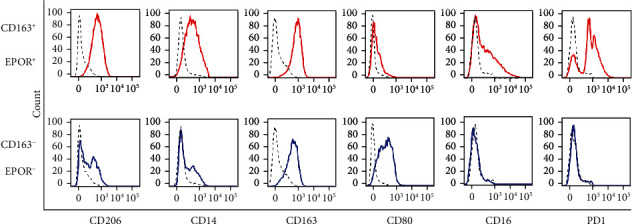
Expression of M1/M2 markers on CD163^+^EPOR^+^ macrophages and CD163^+^EPOR^−^ macrophages in osteosarcoma lung metastasis specimens. Representative flow cytometry analyses revealing differential expression of indicated surface markers on the osteosarcoma CD163^+^EPOR^+^macrophages (upper panel) and CD163^+^EPOR^−^ macrophages (lower panel).

Previous studies have indicated that several M1/M2 surface markers are expressed on macrophages, including CD14, CD16, CD163, CD206, CD80, and PD-1 [9–13, 42]. Having shown that the percentage of CD163^+^EPOR^+^ macrophages was increased in osteosarcoma lung metastasis specimens, we examined the expression profiles of these molecules on CD163^+^EPOR^+^ and CD163^+^EPOR^−^ macrophages in osteosarcoma lung metastasis tissues by flow cytometry.  Figure 4 (upper panel) demonstrates that all CD163^+^EPOR^+^ macrophages were CD14^+^, CD206^+^, and CD163^+^, and a proportion of them expressed CD16 (~32%) and PD1 (~80%), while none expressed CD80.  Figure 4 (lower panel) indicates that CD163^+^EPOR^−^ macrophages were also CD163^+^; however, the mean fluorescence intensity (MFI) of CD163 on CD163^+^EPOR^+^ macrophages is approximately two times compared to CD163^+^EPOR^−^ macrophages. In contrast, CD163^+^EPOR^−^ macrophages did not express CD16 and PD1, and a portion expressed CD14 (~25%) and CD206 (~40%). Moreover, all the CD163^+^EPOR^−^ macrophages expressed CD80. These results indicated that CD163^+^EPOR^+^ macrophages were immunophenotypically distinct from CD163^+^EPOR^−^ macrophages in human osteosarcoma lung metastasis tissues. TAMs are often thought to polarize towards an anti-inflammatory M2 phenotype under tumor microenvironmental stimuli. Our flow cytometry analysis showed that CD163^+^EPOR^+^ macrophages expressed most of the M2 surface markers, whereas CD163^+^EPOR^−^ macrophages expressed an M1 phenotype. Taken together, our data indicated that CD163^+^EPOR^+^ macrophages, but not CD163^+^EPOR^−^ macrophages were TAMs in osteosarcoma lung metastasis specimens.

### 3.5. CD163^+^EPOR^+^ TAMs in Osteosarcomas Expressed Higher M2 Cytokines and Surface Markers than Para-Osteosarcomas

**Figure 5:**
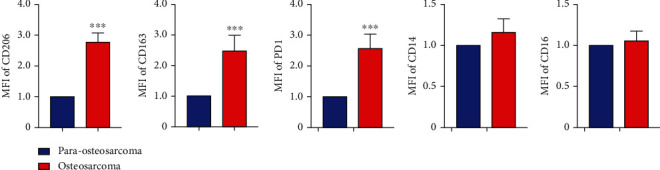
The expression of CD163, CD206, PD1, CD14, and CD16 on CD163^+^EPOR^+^ macrophages and CD163^+^EPOR^−^ macrophages in osteosarcoma and para-osteosarcoma tissues. Quantitative analyses of CD163, CD206, PD1, CD14, and CD16 expression in CD163^+^EPOR^+^ macrophages between osteosarcoma tissues and para-osteosarcoma tissues.

**Figure 6:**
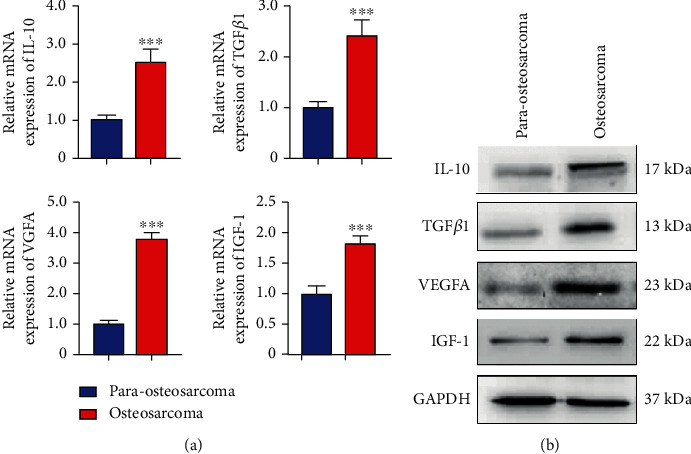
The expression of IL10, TGFB1, VEGFA, and IGF1 on sorted CD163^+^EPOR^+^ TAMs in osteosarcoma tissues and para-osteosarcoma tissues. (a) Quantitative analyses of IL-10, TGF*β*1, VEGFA, and IGF-1 mRNA expression in sorted CD163^+^EPOR^+^ TAMs between osteosarcoma tissues and para-osteosarcoma tissues. (b) Western blot shows IL-10, TGF*β*1, VEGFA, and IGF-1 expression in sorted CD163^+^EPOR^+^ TAMs between osteosarcoma tissues and para-osteosarcoma tissues.

CD163^+^EPOR^+^ macrophages were shown to be true TAMs in osteosarcoma lung metastasis specimens. To develop a further description of CD163^+^EPOR^+^ TAMs in osteosarcoma lung metastasis specimens. We first analyzed the expression profiles of CD163^+^EPOR^+^ TAMs in osteosarcoma and para-osteosarcoma tissues. Our results indicated that the relative mean fluorescence (RMFI) of CD163, CD206, and PD1 was increased in osteosarcoma tissues compared with para-osteosarcoma tissues, while, the expression of CD14 and CD16 did not change (Figure 5). These results indicated that CD163^+^EPOR^+^ TAMs in osteosarcoma tissues expressed higher M2 surface markers than CD163^+^EPOR^+^ TAMs in para-osteosarcoma tissues, which might be due to local environmental stimuli. To further analyze the characterization of CD163^+^EPOR^+^ TAMs in osteosarcomas, we determined M2 cytokine expression of sorted CD163^+^EPOR^+^ TAMs in osteosarcoma and para-osteosarcoma tissues. Our RT-PCR results showed that *IL-10*, *TGF-β1*, *VEGFA*, and *IGF-1* were significantly increased in osteosarcoma tissues (Figure 6(a)). Then, we confirmed the expression of IL-10, TGF-*β*1, VEGFA, and IGF-1 by Western blot.  Figure 6(b) indicated that the protein levels of IL-10, TGF-*β*1, VEGFA, and IGF-1 were significantly increased in osteosarcoma tissues, which was consistent with the RT-PCR results. Collectively, these data indicated that CD163^+^EPOR^+^ TAMs expressed higher M2 cytokines and surface markers in osteosarcoma tissues.

### 3.6. EPO Increased the Expression of M2 Cytokines

**Figure 7:**
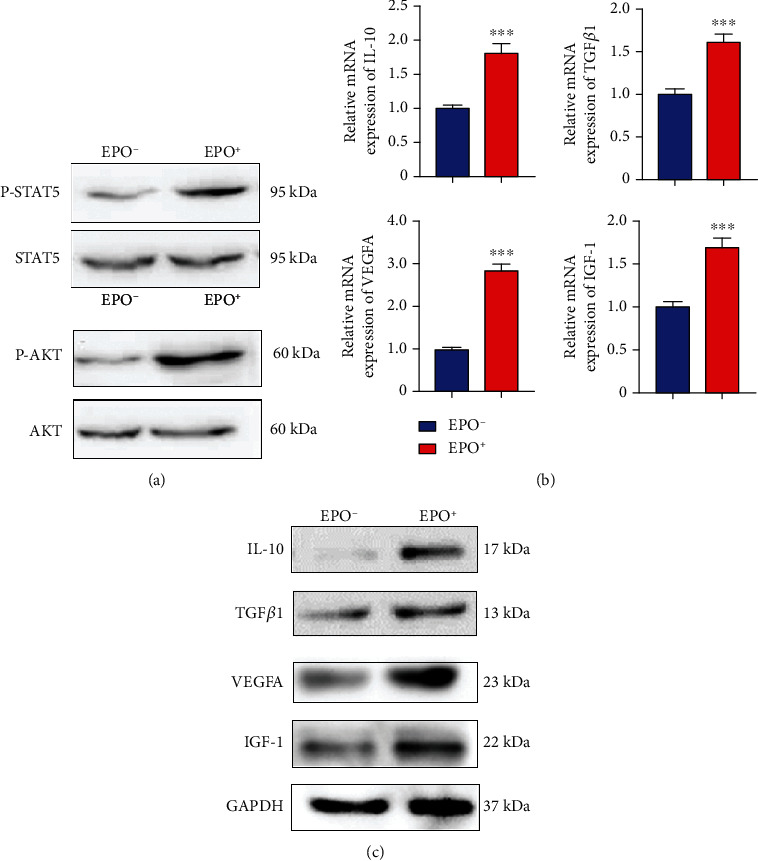
EPO increased the expression of M2 cytokines. (a) Western blot analysis showing phosphorylation of STAT5 and AKT in primary osteosarcoma macrophages upon EPO stimulation. (b) The mRNA expression of IL-10, TGF*β*1, VEGFA, and IGF-1 on osteosarcoma macrophages upon EPO stimulation. (c) The protein expression of IL-10, TGF*β*1, VEGFA, and IGF-1 on osteosarcoma macrophages upon EPO stimulation.

Because most osteosarcoma patients have anemia, which can induce high levels of EPO [17] and TAMs in osteosarcomas express EPOR, verify that EPO plays a role in the protumor function of TAMs, we cultured primary TAMs collected from patients with osteosarcoma lung metastases using CD14 microbeads. The expression of EPOR in TAMs was further verified by showing that EPO stimulation phosphorylates STAT5 and AKT (Figure 7(a)). We then examined the mRNA and protein levels of M2 cytokines after EPO treatment. EPO significantly increased the expression of M2 cytokines (Figures 7(b) and 7(c)). These findings document that EPO/EPOR mediated signaling in TAMs enhanced the expression of M2 cytokines, which can promote the progression of osteosarcoma lung metastases.

### 3.7. CD163^+^EPOR^+^ TAMs Are Associated Closely with Clinicopathologic Factors in Osteosarcoma Lung Metastases

Having shown that TAMs express EPOR and EPO can enhance the proliferation and protumor functions of EPOR^+^ TAMs, we next studied the correlation between the percentage of CD163^+^EPOR^+^ TAMs and clinicopathologic properties. The 106 cases were divided into 2 groups (≥2.5-fold and <2.5-fold) based on the percentage of CD163^+^EPOR^+^ TAMs. As summarized in Table 1, a ≥2.5-fold increase in infiltration of CD163^+^EPOR^+^ TAMs was associated with malignant tumor phenotypes, such as the number of lung metastases, maximal diameter of lung metastases and pathologic grade as well as anemia.

### 3.8. A ≥2.5-Fold Increase in the Percentage of CD163^+^EPOR^+^TAMs in Osteosarcoma Lung Metastases Had a Poor DFS and OS

**Figure 8:**
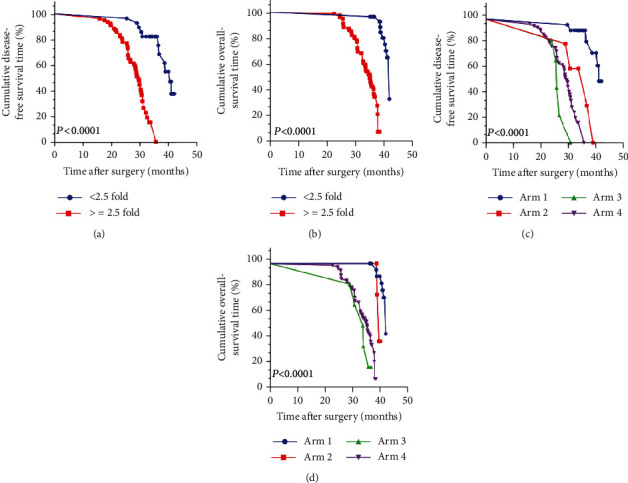
Kaplan–Meier curves for DFS and OS of osteosarcoma lung metastasis according to the percentage of CD163^+^EPOR^+^ TAMs (≥2.5-fold and <2.5-fold) and anemia. *N* = 106. (a) The DFS curves based on the median relative percentage of CD163^+^EPOR^+^ TAMs. (b) The OS curves based on the median relative percentage of CD163^+^EPOR^+^ TAMs. (c) The DFS curves based on Arms 1-4. (d) The OS curves based on Arms 1-4. Note: one blue circle in the blue line is one censored patient and, red square in the red line is one censored patient, one green triangle in the green line is one censored patient, and one purple triangle in the purple is one censored patient.

We further examined the clinical prognosis of the percentage of CD163^+^EPOR^+^ TAMs in the 106 patients with osteosarcoma lung metastases. Compared with the <2.5-fold increase in the percentage of CD163^+^EPOR^+^ TAMs in osteosarcoma lung metastasis patients, the ≥2.5-fold increase in the percentage of CD163^+^EPOR^+^TAMs in osteosarcoma lung metastasis patients had a significantly shorter mDFS and mOS (40.5 vs. 29.4 months, *P* < 0.0001; and 42.0 vs. 35.2 months, *P* < 0.0001, respectively) (Figure 8(a) and Figure 8(b)). As summarized in Table 2, univariate analyses proposed that a ≥2.5-fold increase in the percentage of CD163^+^EPOR^+^ TAMs was significantly associated with decreased mDFS and mOS in osteosarcoma lung metastasis patients. Moreover, multivariate analyses suggested that apart from the number of lung metastases, the maximal diameter of lung metastases, the pathologic grade and anemia, infiltration of ≥2.5-fold increase in the percentage of CD163^+^EPOR^+^ TAMs was still considered an independent prognostic factor in osteosarcoma lung metastases for both mDFS and mOS (hazard ratio [HR] = 2.3, *P* < 0.0001 and HR = 1.6, *P* = 0.003). Collectively, our data implied that the percentage of CD163^+^EPOR^+^ TAMs was a potential significant prognostic factor in patients with osteosarcomas.

### 3.9. Integrated Analyses of Anemia and the Percentage of CD163^+^EPOR^+^ TAMs Provided more Powerful Prognostic Value in Osteosarcoma Lung Metastasis Patients

Because EPOR was expressed by TAMs and anemia can induce EPO secretion, which can activate the EPO-EPOR signal to promote the protumor functions of CD163^+^EPOR^+^ TAMs. Thus, we speculated that combined analyses of anemia and the percentage of CD163^+^EPOR^+^ TAMs may better predict the prognosis of osteosarcoma lung metastasis patients. Based on anemia and the percentage of CD163^+^EPOR^+^ TAMs, patients were classified into four arms, as follows: Arm 1, no anemia, and the percentage of CD163^+^EPOR^+^TAMs < 2.5; Arm 2, anemia, and the percentage of CD163^+^EPOR^+^TAMs < 2.5; Arm 3, no anemia, and the percentage of CD163^+^EPOR^+^TAMs ≥ 2.5; Arm 4, anemia, and the percentage of CD163^+^EPOR^+^TAMs ≥ 2.5; The mDFS for Arms 1-4 were 41.0, 36.7, 25.7, and 29.6 months, respectively (Figure 8(c)); The mOS for Arms 1-4 were 42.0, 39.6, 33.8, and 35.3 months, respectively (Figure 8(d)). Comparing Arms 2-4 and Arm 1, there were significant differences in DFS and OS (*P* < 0.0001). Taken together, our data clearly suggest that combined analyses of anemia and the percentage of CD163^+^EPOR^+^ TAMs were a better predictor for recurrence and survival in osteosarcoma lung metastasis patients than analyzing individual factors.

## 4. Discussion

Previous studies have indicated that many immune cell types form the tumor immune microenvironment, which plays a critical role in cancer progression [43–45]. Specifically, TAM is a main cellular type that infiltrates many cancers, including osteosarcomas [28, 29, 46–49]. TAMs promote proliferation, invasion, and metastasis of tumor cells by stimulating tumor angiogenesis, inhibiting antitumor immune response, and secreting anti-inflammatory cytokines [50]. In this study, we first determined that nearly all the lung tissue-resident macrophages expressed EPOR-eGFP in mice and EPO injection enhanced the proliferation of EPOR^+^ macrophages. Then, we showed that CD163^+^EPOR^+^ macrophages in osteosarcoma lung metastasis specimens are TAMs that have developed a distinctive effect in supporting the progression of osteosarcoma lung metastasis. Several lines of evidence can support this conclusion, as follows: (1) the percentage of CD163^+^EPOR^+^ macrophages increased in osteosarcoma lung metastasis specimens; (2) CD163^+^EPOR^+^ macrophages highly expressed M2 surface marker profile, which is the true TAMs in osteosarcoma lung metastasis; (3) CD163^+^EPOR^+^ TAM numbers are highly associated with poor prognosis of osteosarcoma lung metastasis specimens; (4) sorted CD163^+^EPOR^+^ TAMs highly expressed M2 cytokines, which can promote the progression of osteosarcoma lung metastasis patients; and (5) EPO treatment in primary osteosarcoma lung metastasis macrophages enhanced M2 cytokines expression.

The EPO/EPOR signaling pathway is indispensable for the survival of colony-forming unit-erythroid (CFU-E) and proerythroblasts [51–53]. Growing evidence indicates that EPOR is also expressed in nonerythroid cells, such as kidneys, heart, brain, and various tumor cell lines [20–23]. Specifically, EPOR is also expressed by macrophages in BM, SP, FL, liver, and peritoneum [24–27]. Previous studies indicated that CD163 is a marker of TAMs [28, 29]. In the present study, we determined the expression of CD45, CD163, and EPOR in 106 patients by flow cytometry. Based on the expression of CD163, there were two subsets of TAMs (CD163^+^EPOR^+^ and CD163^+^EPOR^−^). Additionally, M1/M2 marker expression profiles of CD163^+^EPOR^+^ TAMs suggested a distinctive effect in supporting cancer progression. CD206, CD163, and PD1, which are known to be significant for TAM function, were highly expressed in CD163^+^EPOR^+^ TAMs compared with CD163^+^EPOR^−^ TAMs. Taken together, we demonstrated that TAMs in osteosarcoma lung metastasis specimens were characterized by the expression of EPOR, suggesting that EPO may play a pivotal role not previously illustrated.

This study showed that >2.5-fold percentage of CD163^+^EPOR^+^ TAMs in osteosarcoma tissues had a significant impact on osteosarcoma prognosis. Previous studies indicated that CD163^+^TAM infiltration has a poor prognosis in many cancers [28, 29]. We quantified the relative percentage of CD163^+^EPOR^+^ TAMs in osteosarcoma lung metastasis tissues. The percentage of CD163^+^EPOR^+^ TAMs increased 2.5-fold, while the percentage of CD163^+^EPOR^−^ TAMs was not significantly different. Our results further emphasized that CD163^+^EPOR^+^ TAMs are associated with the progression of osteosarcoma lung metastasis.

TAMs enhance the progression of osteosarcoma lung metastasis patients, but the molecular mechanisms have not been thoroughly studied. Our findings show that M2 cytokines were highly expressed by CD163^+^EPOR^+^ TAMs in osteosarcoma lung metastasis tissues suggested the possibility that CD163^+^EPOR^+^ TAMs enhanced the progression of osteosarcoma lung metastasis patients by secreting many different M2 cytokines. Of note, IGF-1 increases the proliferation of many cancer cells [54–56], IL-10, and TGF-*β*1 inhibits the immune responsiveness of T cells [57, 58], and VEGFA enhances angiogenesis [59, 60]. Our results further illuminate the contributions of TAMs in the progression of osteosarcoma lung metastasis patients and research on detailed mechanisms are warranted in the future.

A number of mechanisms of EPO involvement in cancer progression have been proposed. EPO increases the expression of EPOR in cancer cells, thereby increasing cancer cell growth and metastasis [61]. EPO directly increases the vascular angiogenesis of cancer cells and EPO increases the macrophage secretion of VEGFC, then increases lymphangiogenesis and nodal metastasis [61, 62]. In the present study, we showed that EPO enhances the expression of M2 cytokines by TAMs in osteosarcoma lung metastasis patients, which led to the progression of cancers by different mechanisms. These results suggested that the expression of M2 cytokines by TAMs induced by EPO may be a new mechanism underlying cancer progression.

Most osteosarcoma patients have anemia, which can induce high levels of EPO [17]. Generally, osteosarcoma with anemia is correlated with a worse prognosis. In a previous study, anemia was reported to induce an increased immunosuppressive CD45^+^ EPC population, which promotes cancer progression [63]. Our study indicated that anemia in combination with a >2.5-fold increase in the percentage of CD163^+^EPOR^+^ TAMs had the worst prognosis. Anemia leads to increased EPO concentration [64]. In our study, we injected EPO into mice and found that the infiltration of lung EPOR^+^ macrophages increased significantly. Thus, to some extent, this finding might provide a potential new mechanism for the poor prognosis of osteosarcoma patients with anemia.

**Figure 9:**
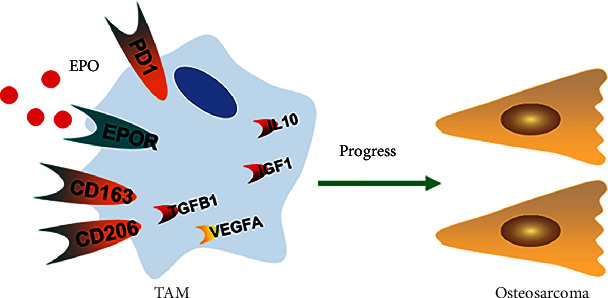
Schematic diagram of EPOR+ TAMs in the progression of osteosarcoma patients.

In conclusion, we have identified and characterized CD163^+^EPOR^+^ macrophages as TAMs in osteosarcoma lung metastasis patients. Our data demonstrated that the increased percentage of CD163^+^EPOR^+^ TAMs was associated with a poor prognosis for osteosarcoma lung metastasis patients. Further studies indicated that EPO enhanced tumor progression by increasing the infiltration of CD163^+^EPOR^+^ TAMs and expression of M2 cytokines (schematic in Figure 9). In the future, RNA-seq analyses of CD163^+^EPOR^+^ TAMs may help us to better understand of this subset of TAMs, and targeting TAM therapy may be a potential therapeutic intervention in osteosarcoma lung metastasis patients.

## Figures and Tables

**Table 1:** Association between the percentage of CD163^+^EPOR^+^ TAMs variables and clinical pathological properties of patients with osteosarcoma lung metastasis in this study (*N* = 106).

Characteristics	CD163^+^EPOR^+^ TAMs		*χ* ^2^	*P* value
	≥2.5	<2.5		
Age (years)				
<25	57	21		
≥25	22	6	0.328	0.567
Gender				
Male	52	18		
Female	27	9	0.006	0.936
Location of primary tumor				
Femur and tibia	73	22		
Other	6	5	2.582	0.108
Size of primary tumor (cm)				
≥5	40	13		
<5	39	14	0.05	0.824
The number of lung metastases				
Multiple	48	10		
Single	31	17	4.57	0.033
Maximal diameter of lung metastases				
≥5	63	5		
<5	16	22	32.803	0.000
Pathologic grade				
Well to moderately	13	19		
Poorly to undifferentiated	66	8	27.754	0.000
Anemia				
Yes	73	5		
No	6	22	56.516	0.000

**Table 2:** Univariate and multivariate analyses of prognostic factors correlated to DFS and OS of patients with osteosarcoma lung metastases in this study (*N* = 106).

Variables	DFS				OS			
	Univariate		Multivariate		Univariate		Multivariate	
	*P*	HR	95% CI	*P*	*P*	HR	95% CI	*P*
Age (years) (<25 VS. ≥25)	0.196	—	—	—	0.285	—	—	—
Gender (male VS. female)	0.880	—	—	—	0.4703	—	—	—
Location of primary tumor (femur and tibia VS. other)	0.432	—	—	—	0.809	—	—	—
Size of primary tumor (cm) (≥5 VS. <5)	0.665	—	—	—	0.651	—	—	—
The number of lung metastases (multiple VS. <single)	<0.0001	2.7	1.62-4.2	<0.001	<0.0001	1.63	1.2-2.7	0.007
Maximal diameter of lung metastases (≥5 VS. <5)	0.060	—	—	—	0.070	—	—	—
Pathologic grade (well to moderately VS. poorly to undifferentiated)	<0.0001	3.4	1.6-6.9	0.001	<0.0001	2.3	1.3-4.1	0.006
Anemia (yes VS. no)	<0.0001	2.4	1.8-4.5	<0.001	<0.0001	1.8	1.2-2.65	0.005
The percentage of TAMs (≥2.5 VS. <2.5)	<0.0001	2.3	1.7-4.3	<0.001	<0.0001	1.6	1.1-2.7	0.003

## Data Availability

All data generated in the study are included in the present article and its supplementary information files.

## Supplementary Materials

Supplementary Materials Supplementary Figure 1: BrdU and GFP-FMO were as negative control to gate negative and positive populations. (A) Representative flow cytometry analyses showed that no BrdU injection mice have no BrdU expression. (B) Representative flow cytometry analyses showed that no GFP expression in the negative control.
