# Supplementary material for: Identification and Functional Analysis of EPOR+ Tumor-Associated Macrophages in Human Osteosarcoma Lung Metastasis
Source: J Immunol Res. 2020 Aug 18;2020:9374240. doi: 10.1155/2020/9374240 (PMC7450330; doi:10.1155/2020/9374240)
Supplement: Supplementary Materials — Supplementary Figure 1: BrdU and GFP-FMO were as negative control to gate negative and positive populations. (A) Representative flow cytometry analyses showed that no BrdU injection mice have no BrdU expression. (B) Representative flow cytometry analyses showed that no GFP expression in the negative control. [file 9374240.f1.pptx]

## Slide 1
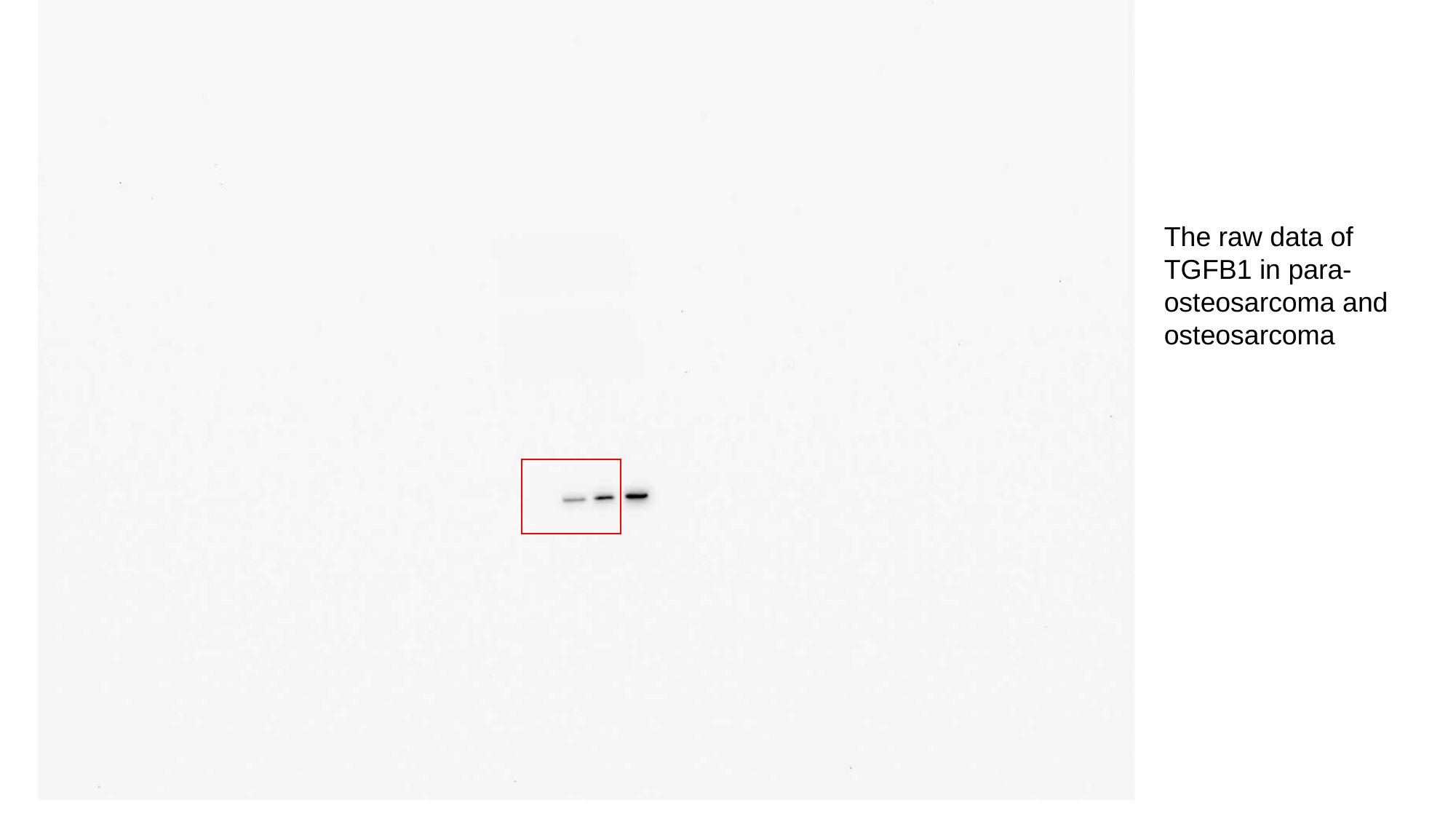

The raw data of TGFB1 in para-osteosarcoma and osteosarcoma

## Slide 2
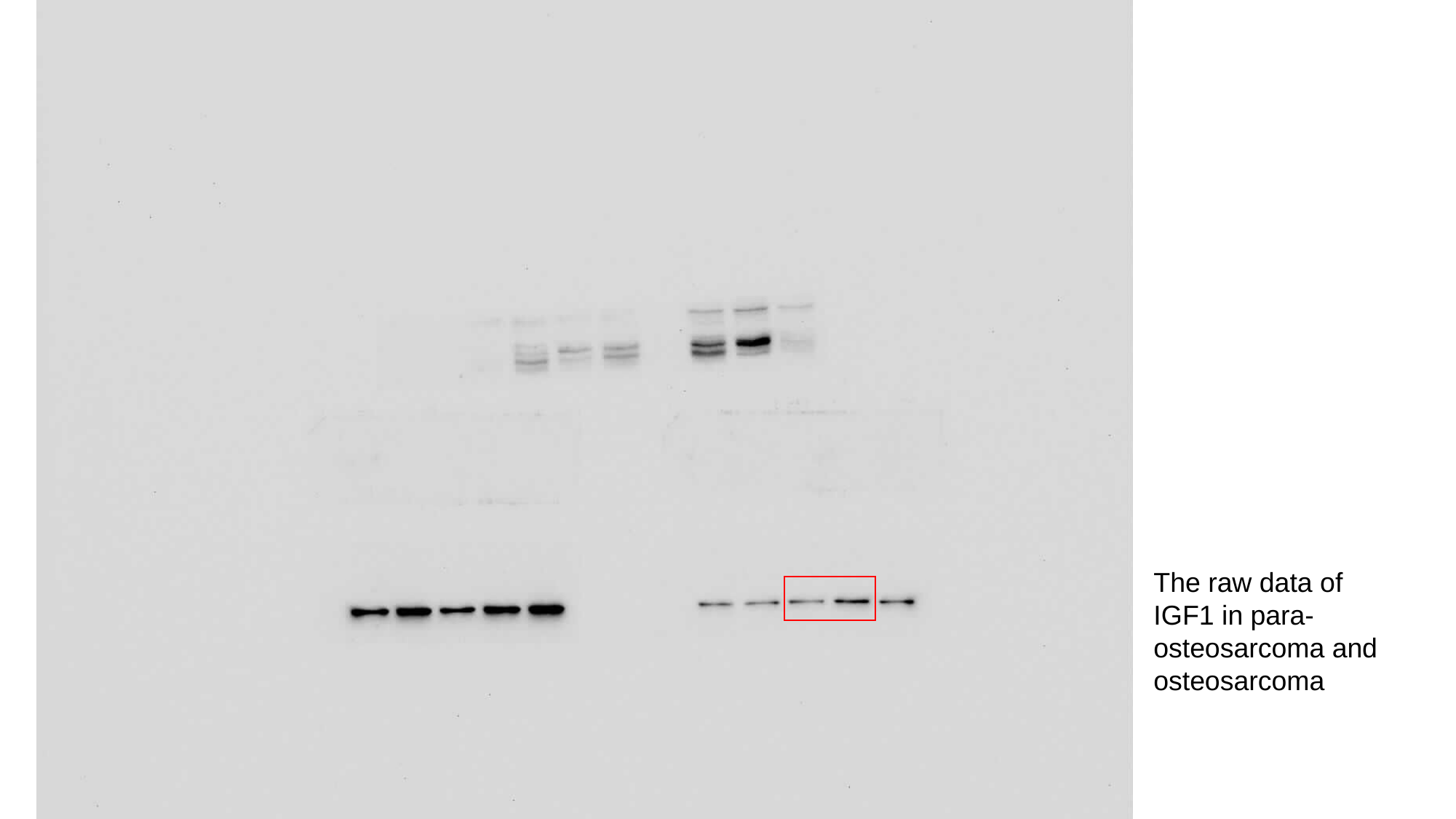

The raw data of IGF1 in para-osteosarcoma and osteosarcoma

## Slide 3
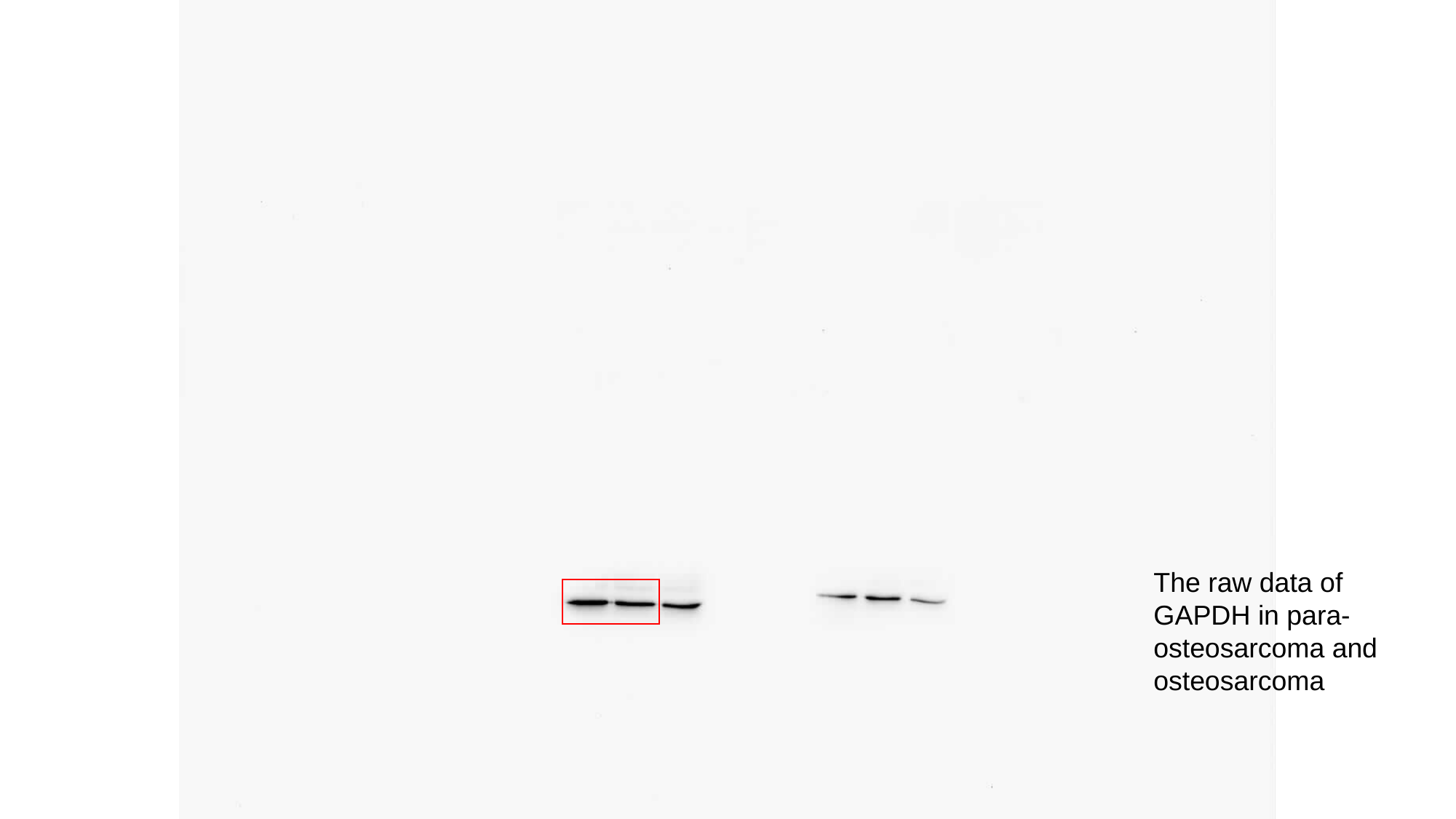

The raw data of GAPDH in para-osteosarcoma and osteosarcoma

## Slide 4
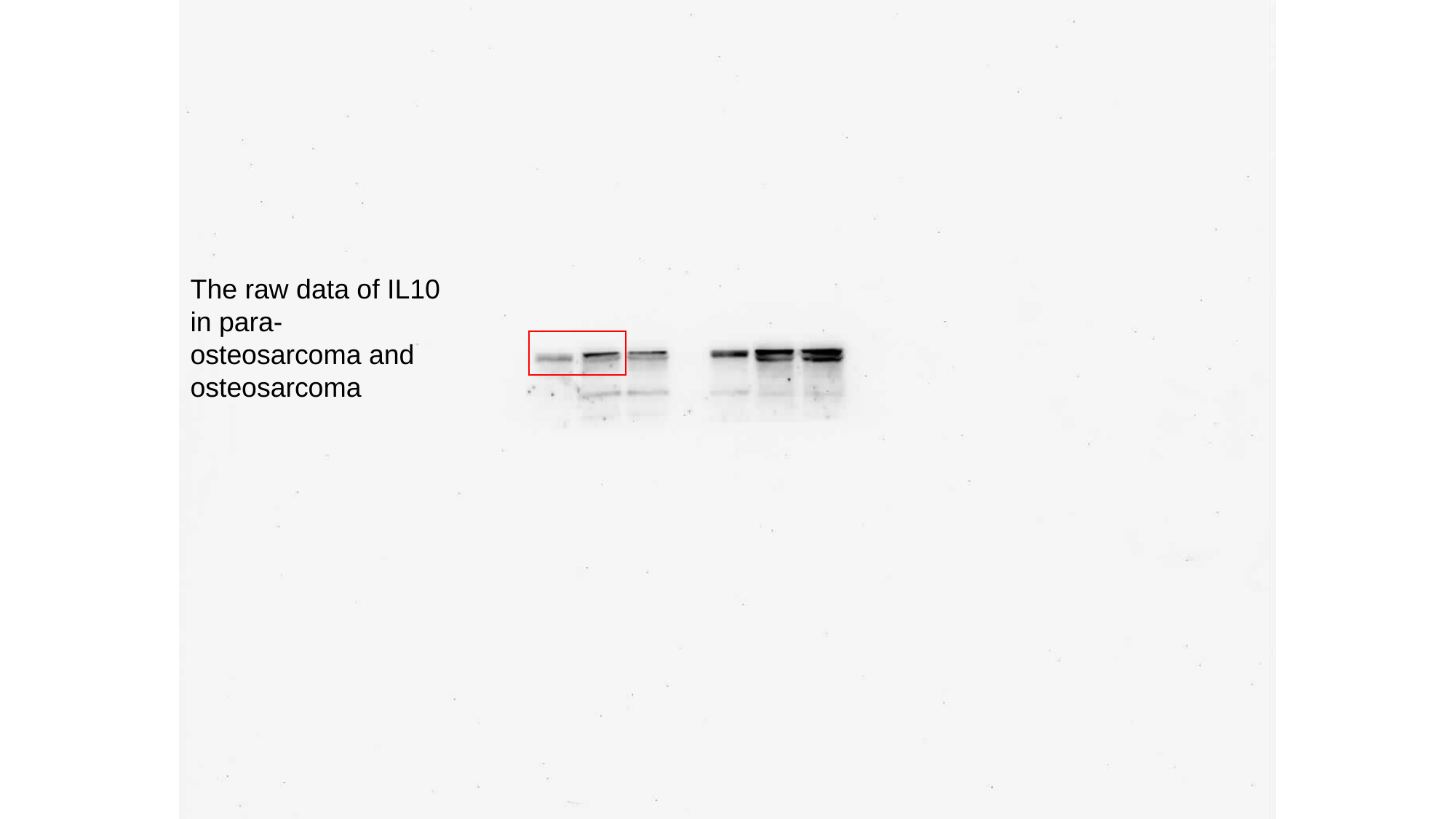

The raw data of IL10 in para-osteosarcoma and osteosarcoma

## Slide 5
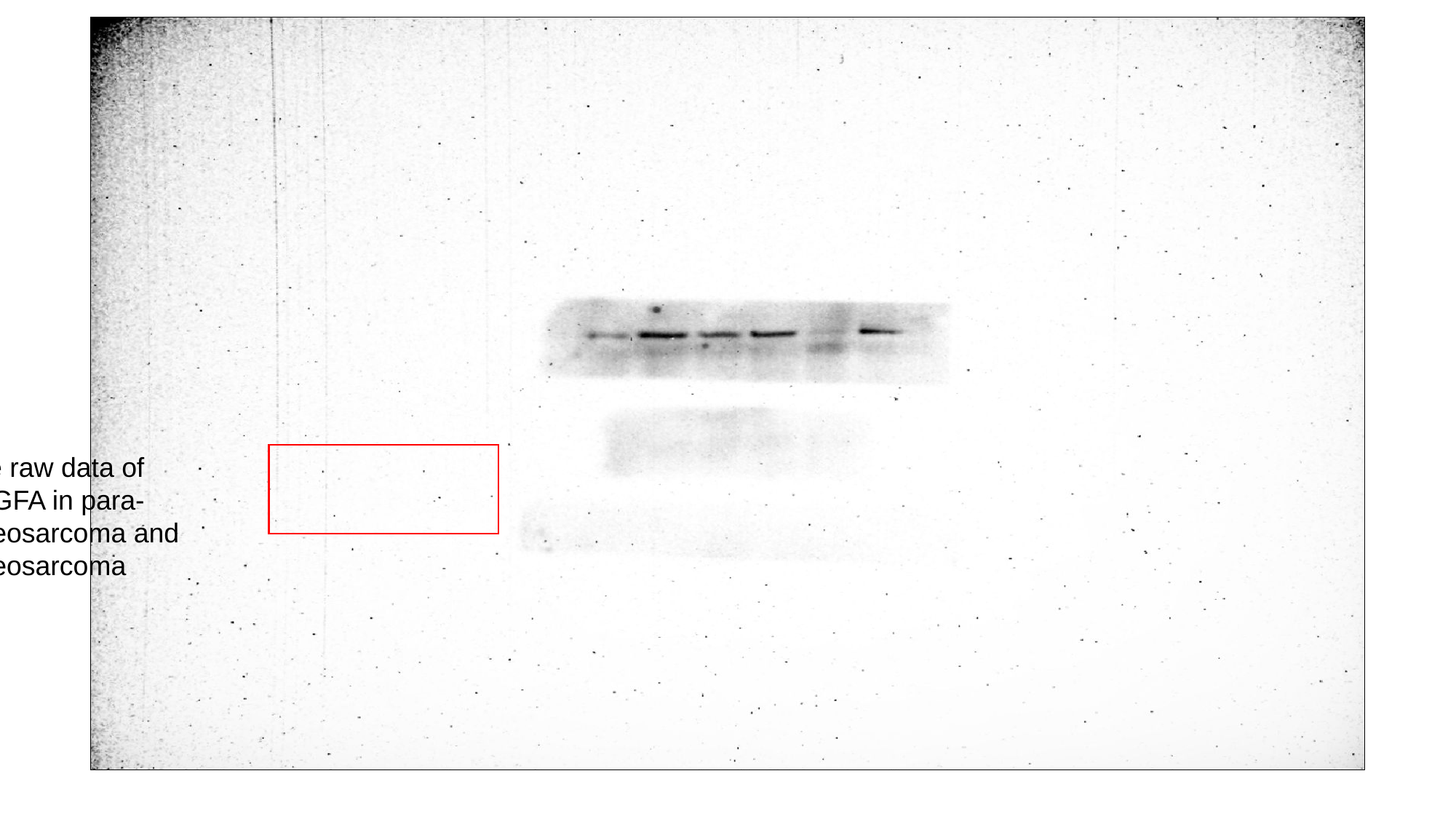

The raw data of VEGFA in para-osteosarcoma and osteosarcoma

## Slide 6
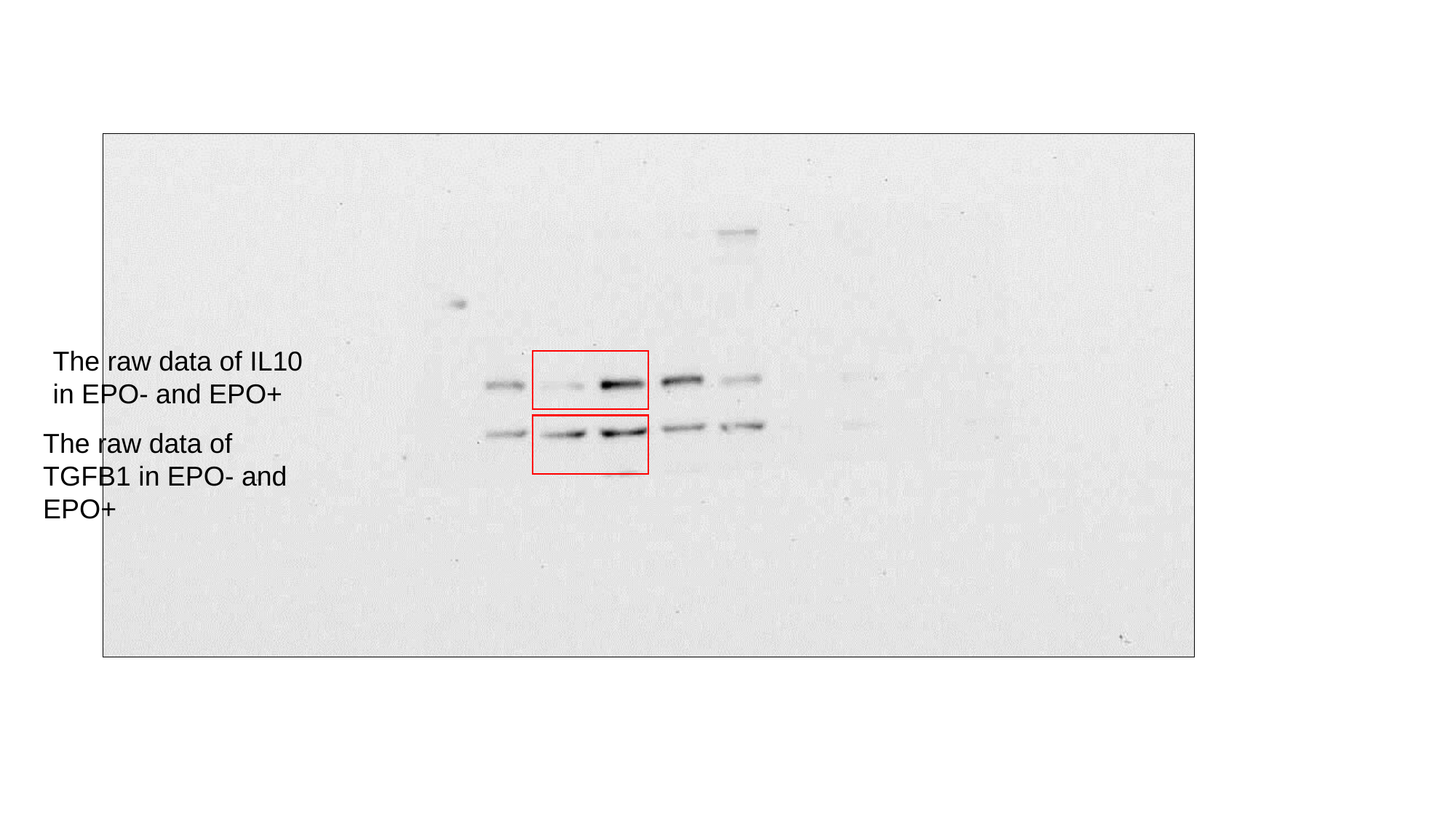

The raw data of IL10 in EPO- and EPO+
The raw data of TGFB1 in EPO- and EPO+

## Slide 7
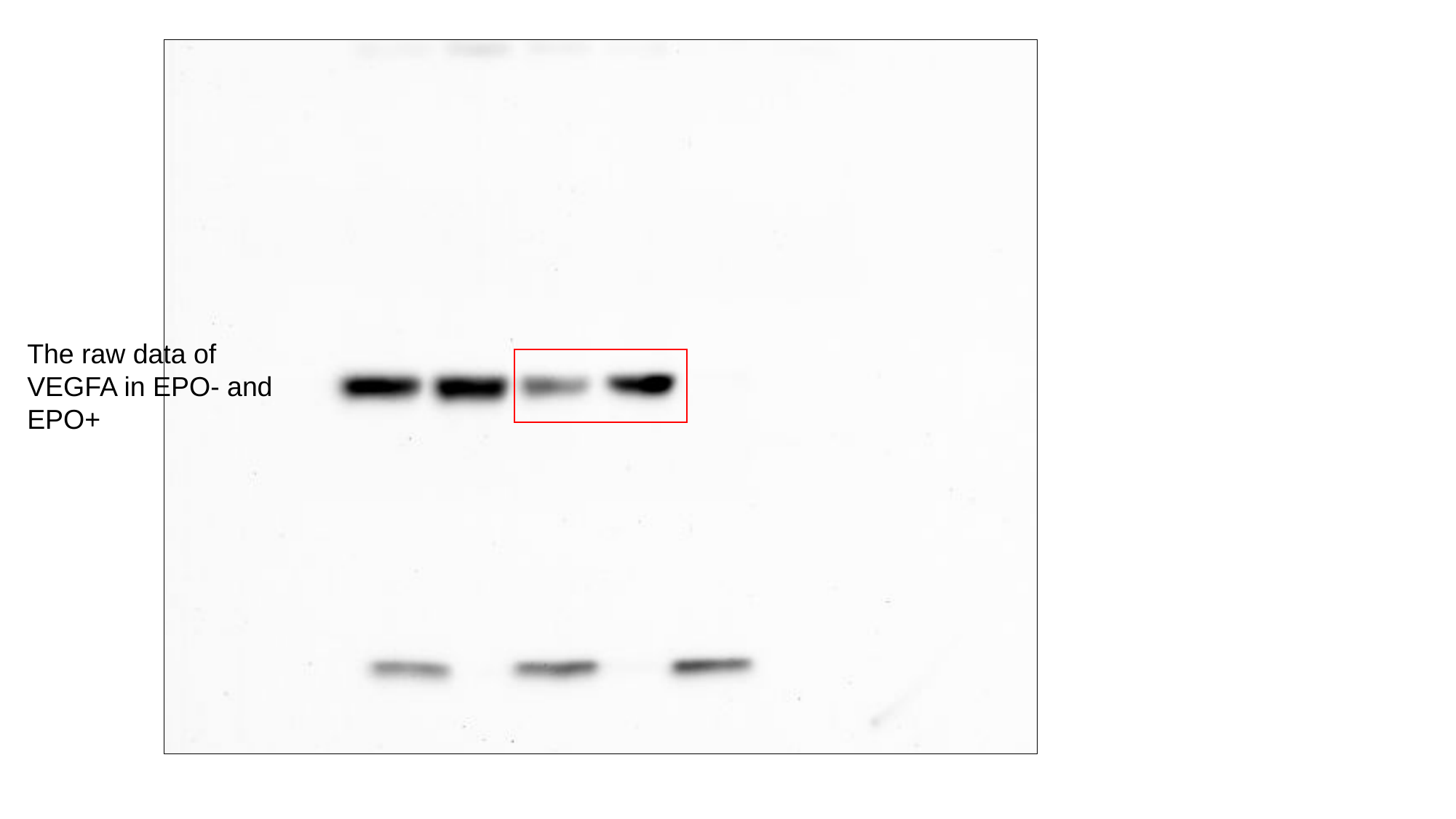

The raw data of VEGFA in EPO- and EPO+

## Slide 8
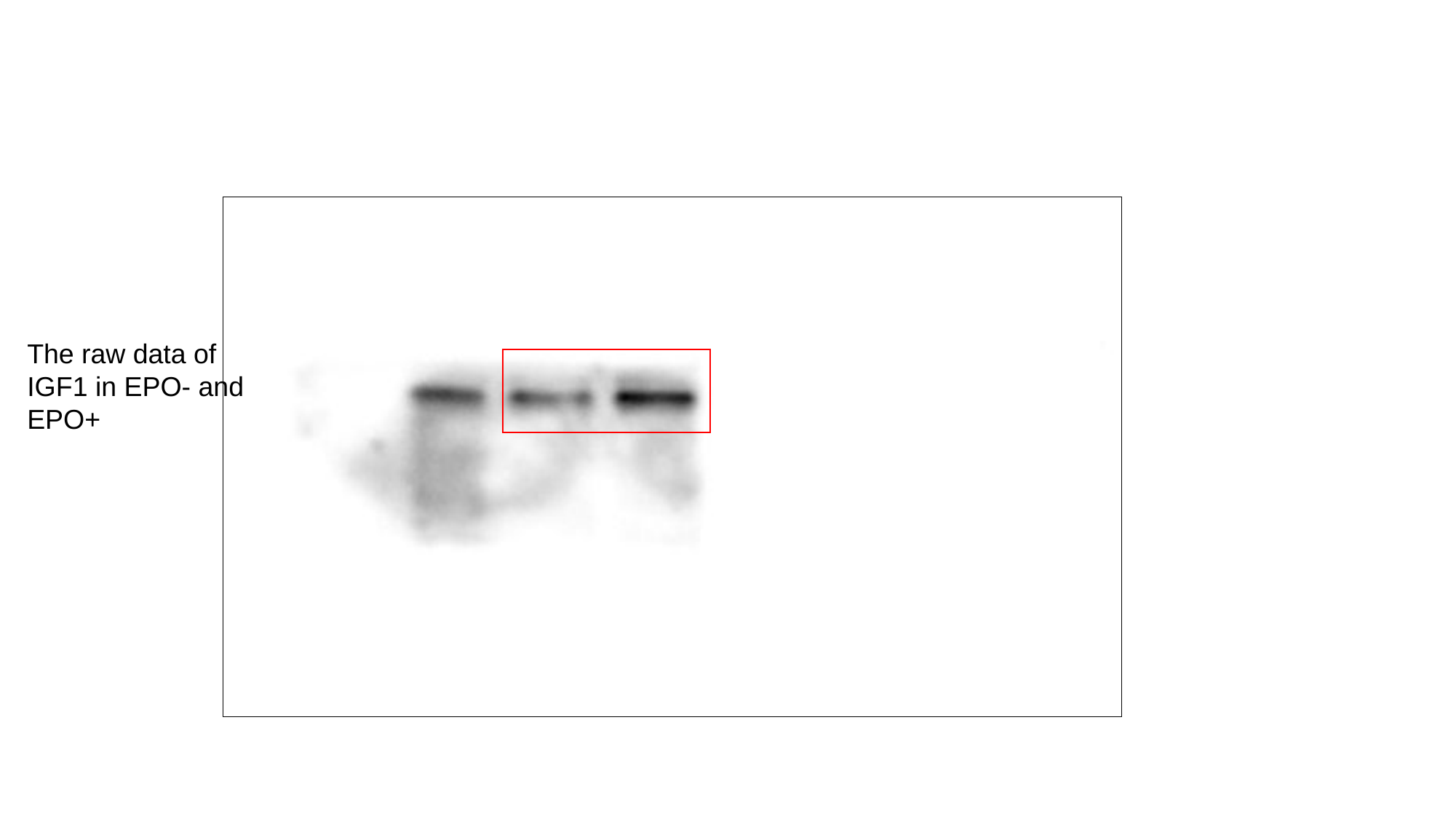

The raw data of IGF1 in EPO- and EPO+

## Slide 9
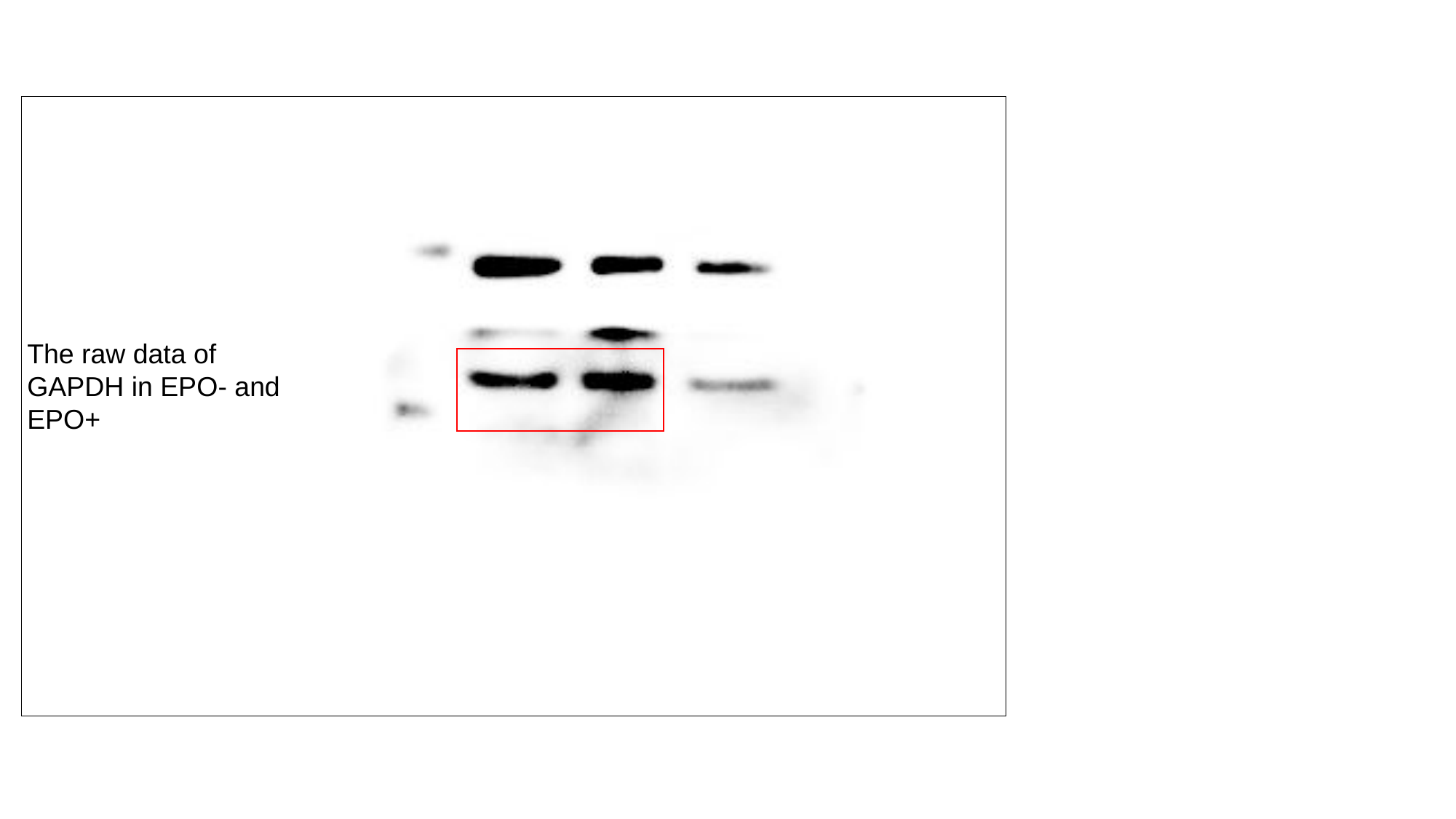

The raw data of GAPDH in EPO- and EPO+
